# Polarization-Dependent Coding Metasurface with Switchable Transmission and RCS Reduction Bands

**DOI:** 10.3390/mi14010078

**Published:** 2022-12-28

**Authors:** Hamza Asif Khan, Chenxi Huang, Qiang Xiao, Syed Muzahir Abbas

**Affiliations:** 1 Institute of Electromagnetics Space, Southeast University, Nanjing 210096, China; 2 State Key Laboratory of Millimeter Waves, Southeast University, Nanjing 210096, China; 3 School of Engineering, Faculty of Science and Engineering, Macquarie University, Sydney, NSW 2109, Australia

**Keywords:** coding metasurface, radar cross-section (RCS) reduction, switchable, transmission, reflection, diffusion

## Abstract

In this article, a coding metasurface is specifically designed to switch transmission and reflection functionalities between two different frequency bands for linearly polarized waves within wide incidence angles. A metasurface consists of four metallic patterns, where the middle two structures are inserted to ensure effective performance of transmission and reflection, while the top and bottom patterns are designed based on simultaneously controlling the reflection phase for both polarization states. It has been experimentally demonstrated that the proposed metasurface can convert a transmission band into a complete reflection band (meanwhile, the reflection band is translated into a complete transmission band) by changing the incident polarization state. Highly efficient transmission and reflection characteristics have been achieved from 21.1 to 24.5 GHz as well as from 33.3 to 38.3 GHz, whereas more than 10 dB radar cross-section (RCS) reduction has also been obtained for both TE and TM modes in their respective reflection bands. The performance of the proposed metasurface is well sustained up to 40° oblique incidence. This work will help to open a new aspect in metasurfaces to manipulate the electromagnetic waves at preferable frequency bands to achieve desirable functionalities.

## 1. Introduction

In military systems, radar is a key component for the detection of combatant targets [1]. With the rapid advancements in radar detection technology, it becomes more challenging to escape radar without being detected. To solve this problem, military stealth technology is deployed [2]. The main objective of military stealth technology is to minimize radar cross section (RCS), so it can prevent the target from being detected [3]. Mainly two techniques are used to perform RCS reduction [4]. The first approach is by using radar absorbing material (RAM) that converts the EM energy into heat that causes the rise in temperature and might be detected by infrared detectors. Another approach for RCS reduction is by scattering the reflected EM waves away from the direction of the source [5]. Both absorption and scattering can be achieved with the help of metasurfaces.

Metasurfaces (MS), which are the 2D counterpart of metamaterials, consist of subwavelength unit cells [6,7,8]. These structures are light in weight, low cost, and easy to fabricate. Metasurfaces attain a considerable attention in military stealth due to low profile and strong electromagnetic (EM) wave manipulation abilities [9,10,11]. They can easily control the amplitude, polarization, and phase of EM waves [12,13,14]. Recently, it has been observed that metasurfaces have extensively been used to perform absorption and scattering. Landy et al. introduced a perfect absorber for the first time with narrow band characteristics [15]. Later on, efforts have been made to increase the operating bandwidth by designing multilayer structures [16], decoupling optical properties [17], exciting quasi-BIC resonance [18], and frequency selective surface (FSS) loaded with lumped elements [19,20]. However, these structures are not suitable for use practically due to their bulky nature, complex design, and higher cost. Cui et al. initiated the concept of coding metamaterials [21]. Coding MS are designed by the distribution of different coding elements having different phases while the amplitude remains the same. With the correct placement of coding elements on a 2D planar structure, the EM waves can be diffused in multiple directions that will leave minimum scattering energy in all directions, and thus, RCS will be reduced. Ultrawideband [22,23] and multiband [24] RCS reduction are efficiently achieved by using diffusion coding MS. Absorption and diffusion mechanisms have also been combined together to achieve RCS reduction [25,26,27]. However, integrated multifunctional devices are the need of modern application systems. Along with RCS reduction, metadevices must have the capability to perform other functions, such as transmission, to make them more suitable for stealth radomes. Therefore, FSS are deployed in radomes, with spectral filtering properties to achieve a transparent window along with RCS reduction [28]. Later, MS are combined with FSS structures to perform wideband RCS reduction with a transparent window [29,30,31]. Nevertheless, all these reported MS can only work in a transparent mode or reflection mode in a particular band, leaving the performance fixed for all polarizations. Recently, few passive metasurfaces have been reported to switch transmission and reflection characteristics in the same frequency band to achieve controllable performance of EM waves by changing the helicity, frequency, and polarization of incident waves [32,33,34]. However, they are limited to a single working band, where it converts either the reflection into transmission or the transmission into reflection. Moreover, their practical use in modern devices still needs to be discovered especially for wideband RCS reduction.

In this paper, we propose a transmission–reflection switchable coding metasurface that is capable of switching features in dual frequency bands depending on the incident polarization. To illustrate this concept in the passive area, we designed an anisotropic unit cell that is able to switch the transmission and reflection at a particular frequency band based on TE-polarization and TM-polarization. Simulated and experimental results indicate that the designed metasurface has the ability to offer switchable phenomena. Moreover, the simulated and experimental results are in good agreement, which validates the presented concept. This concept can be used to utilize the full space more efficiently.

## 2. Working Principle

First, we will discuss the concept of designing a switchable coding element. For a coding element with mirror symmetry, its EM properties can be defined using Jones matrices.

(1) $$R\left(x,y\right)=\left(\begin{array}{cc} r_{xx,f1}\left(x,y\right) & 0\\ 0 & r_{yy,f2}\left(x,y\right) \end{array}\right)$$

(2) $$T\left(x,y\right)=\left(\begin{array}{cc} t_{xx,f2}\left(x,y\right) & 0\\ 0 & t_{yy,f1}\left(x,y\right) \end{array}\right)$$

Here, transmission coefficients are represented by $t_{xx}$ and $t_{yy}$, while reflection coefficients are described as $r_{xx}$ and $r_{yy}$ along the given axes, respectively. An ideal condition will be considered to ensure high efficiency of transmission and reflection. For the efficient transmission, $\left|t_{xx}\right|=\left|t_{yy}\right|=1$; meanwhile, reflection will be considered zero. Similarly for complete reflection, T = 0; meanwhile, $\left|r_{xx}\right|=\left|r_{yy}\right|=1$. Transmitted and reflected waves must be manipulated together to have switchable phenomena in dual band. For this, Jones matrices need to satisfy the following conditions:

(3) $$\left|r_{xx,f1}\right|=\left|t_{yy,f1}|\&\;\left|r_{yy,f1}\right|=|t_{xx,f2}\right|$$

**Figure 1:**
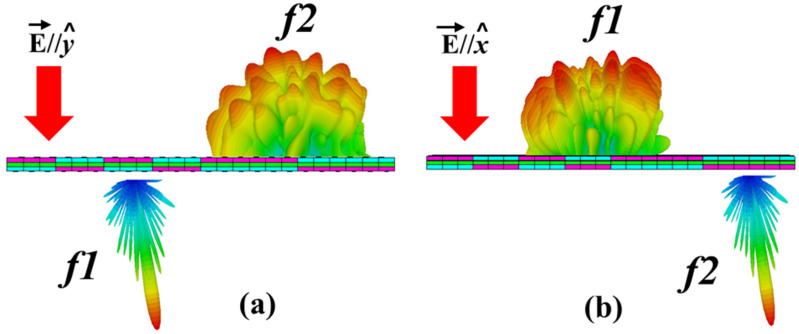
Conceptual illustration of the proposed metasurface for (**a**) the *y*-polarized wave (**b**) the *x*-polarized wave.

According to Equation (3), the metasurface will act as a reflector for the *x*-polarized wave and shows transparent characteristics for the *y*-polarized wave at the *f*1 frequency band, and the complete transparent characteristics for the *x*-polarized wave and the reflected properties for the *y*-polarized wave at the *f*2 band. Both frequency bands must have characteristics to flexibly tune according to the geometric structure. If both the frequency bands are tuned in such a way that they can overlap with each other with high efficiency transmission and reflection characteristics, then we are able to switch the functionalities between two working bands. Figure 1 illustrates the working mechanism of the proposed idea.

Finally, the concept of scattering to achieve RCS reduction is explained. When a normal EM wave strikes the coding metasurface, which consists of an array of *l* × *m* coding elements, then the scattering far fields can be expressed as [29]:

(4) $$F\left(\theta ,\varphi \right)=f_{l,m}\left(\theta ,\varphi \right)S_{n}\left(\theta ,\varphi \right)$$

Here, *θ* and *φ* represent the elevation and the azimuth angles of a reflected wave. $f_{l,m}\left(\theta ,\varphi \right)$ represents the primary pattern (electric field), and $S_{n}\left(\theta ,\varphi \right)$ is an array pattern. Meanwhile, the suffix “n” represents an array of *l* × *m* coding elements in the *x* and *y*-direction. It is a scalar quantity and can be calculated as:

(5) $$S_{n}\left(\theta ,\varphi \right)=\sum_{l=1}^{L}\sum_{m=1}^{M}\exp\left\{j\left[\varphi_{l,m}+k_{0}D_{x}\left(l-1/2\right)\times \left(\sin\theta \cos\varphi -\sin\theta_{i}\cos\varphi_{i}\right)+k_{0}D_{y}\left(m-1/2\right)\left(\sin\theta \cos\varphi -\sin\theta_{i}\sin\varphi_{i}\right)\right]\right\}$$

Using the values of the array pattern in Equation (4), the above equation is expressed as:

(6) $$F\left(\theta ,\varphi \right)=f_{l,m}\left(\theta ,\varphi \right)\sum_{l=1}^{L}\sum_{m=1}^{M}\exp\left\{j\left[\varphi_{l,m}+k_{0}D_{x}\left(l-1/2\right)\times \left(\sin\theta \cos\varphi -\sin\theta_{i}\cos\varphi_{i}\right)+k_{0}D_{y}\left(m-1/2\right)\left(\sin\theta \cos\varphi -\sin\theta_{i}\sin\varphi_{i}\right)\right]\right\}$$

Here, $\varphi_{l,m}$ is the phase response of each super cell. $D_{x}$ and $D_{y}$ are the sizes of the super cell in the direction of the *x* and *y* axes, respectively. $k_{o}$ is the free space vector of the incident wave. Therefore, through the correct placement of coding elements in a metasurface, arbitrary scattering patterns can be achieved.

## 3. Design and Analysis

### 3.1. Coding Element Design

**Figure 2:**
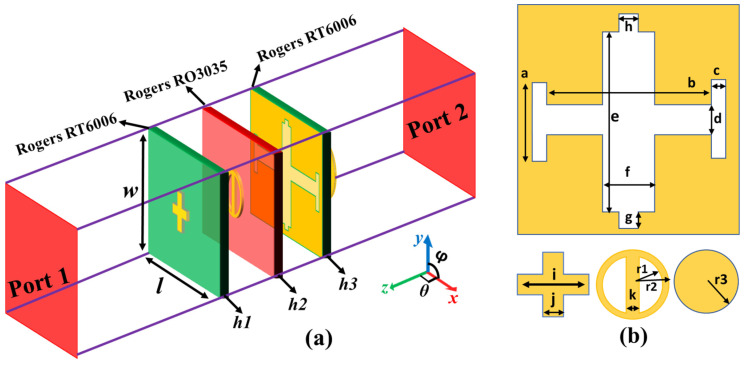
Structures and simulation setup of a unit cell. (**a**) Perspective view with its simulation setup. (**b**) Structure of four metal layers. *l* = 3 mm, *w* = 3.6 mm, *h*1 = 0.8 mm, *h*2 = 0.76 mm, *h*3 = 0.8 mm, a = 0.5 mm, b = 2.3 mm, c = 0.15 mm, d = 0.2 mm, e = 1.7 mm, f = 0.45 mm, g = 0.15 mm, h = 0.15 mm, *i* = 0.8 mm, j = 0.2 mm, k = 0.2 mm, *r*1 = 0.7 mm, *r*2 = 0.5 mm, and *r*3 = 0.5 mm.

To further illustrate the physics of Equation (3), an anisotropic unit cell is presented in Figure 2. This structure exhibits polarization-dependent EM characteristics. It consists of four metallic patterns, which are printed on three dielectric substrate layers. The middle copper layer consists of a Jerusalem cross slot structure, which will behave as a metal for the reflection band and passband for the transparent window. Due to different slot structures in the *x-* and *y*-directions, it permits to tune the resonance frequency. Meanwhile, due to the metal presence in a ringlike structure along the *y*-direction, the incident wave in the *x*-direction can easily penetrate through it with high efficiency. A top pluslike structure and a bottom cylindrical structure are used to tune the reflection phase at desired frequency bands, leaving the reflection amplitude nearly unchanged. The relative permittivity of the *h*1 and *h*3 dielectric substrate is 6.15 and the loss tangent (tanδ = 0.0015) has a thickness of 0.8 mm, while the *h2* dielectric substrate has a relative permittivity and a loss tangent of $\varepsilon_{r}$ = 3.6 and tanδ = 0.0015, respectively, with 0.76 mm of thickness, which is easily available commercially. The thickness of all metallic plates is 0.035 mm with an electric conductivity of $5.8\times 10^{7}S/m$.

**Figure 3:**
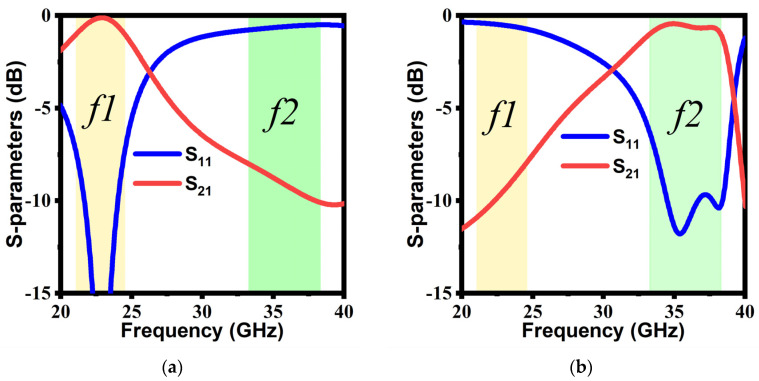
S-parameters: (**a**) TE mode; (**b**) TM mode.

The proposed unit cell was analyzed using CST Microwave Studio to achieve desired functionalities. In the simulation environment, the boundary conditions for *x* and *y* planes were set as a unit cell, and two Floquet ports were installed in +*z* and −*z*-directions. As the proposed structure exhibits anisotropic properties, the unit cell will show the distinguishable response for differently polarized incident EM waves. Figure 3 depicts the transmission and reflection results for transverse electric (TE) and transverse magnetic (TM) polarizations under normal incidence for a unit cell. TE polarization represents an incident wave with its electric field orientation along the *y*-direction, while the orientation of the electric field is along the *x*-direction for the TM polarization.

**Figure 4:**
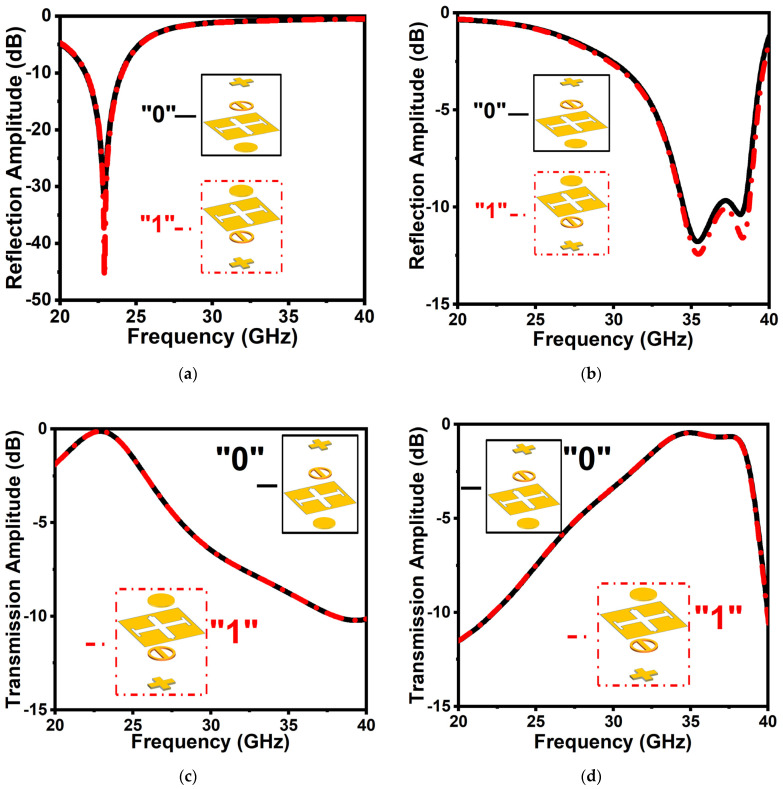
Amplitude of the elements “0” and “1” for reflection: (**a**) TE wave; (**b**) TM wave. Transmission amplitude of (**c**) TE and (**d**) TM waves, respectively.

The element “1” is the same as the element “0” with a rotation of 180° clockwise around the *x*-axis. For the unit cell “0”, port 1 is considered an incidence source to analyze the wavefront, while for the unit cell “1”, port 2 is considered an incidence source. To investigate the reflection and the transmission properties of the proposed coding elements “0” and “1”, first, we will discuss the amplitude under normal illumination of TE and TM waves. As can be seen in Figure 4a, for both coding elements “0” and “1”, a reflection efficiency of more than 90% is realized for a normal incidence TE wave from 33.3 to 38.3 GHz. Simultaneously, for the TM wave, a highly efficient reflection is experienced from 21.1 to 24.5 GHz, as shown in Figure 4b. It can be observed that the reflection amplitude remains higher than −1 dB in dual-frequency bands for both polarization states. Meanwhile, less than 1 dB insertion loss is observed in transmission for the TE wave from 21.1 to 24.5 GHz, and similarly, for the TM wave, a frequency band of 33.3 to 38.3 GHz experienced an insertion loss of less than 1 dB in transmission, as shown in Figure 4c,d, respectively.

**Figure 5:**
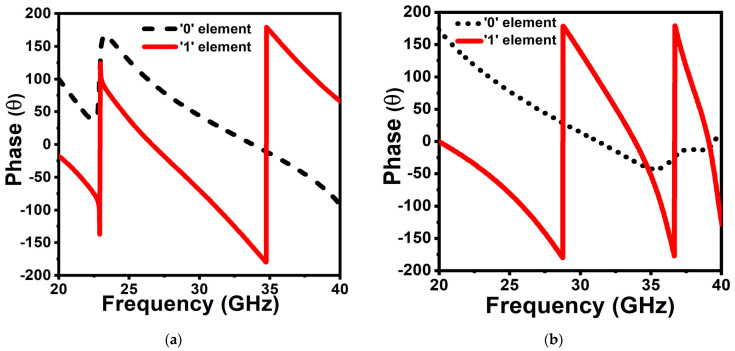
Reflection phase of two elements for (**a**) TE wave and (**b**) TM wave.

Besides, the reflection phases of the elements “0” and “1” under normal TE mode and TM mode are shown in Figure 5a,b, respectively. It is noted that the phase difference between the coding elements for the TE mode is kept nearly π between 33 and 40 GHz, and similarly, the phase difference is restricted to nearly π for the TM mode from 20 to 28.8 GHz.

### 3.2. Arrangement of Checkerboard Metasurface for RCS Reduction

**Figure 6:**
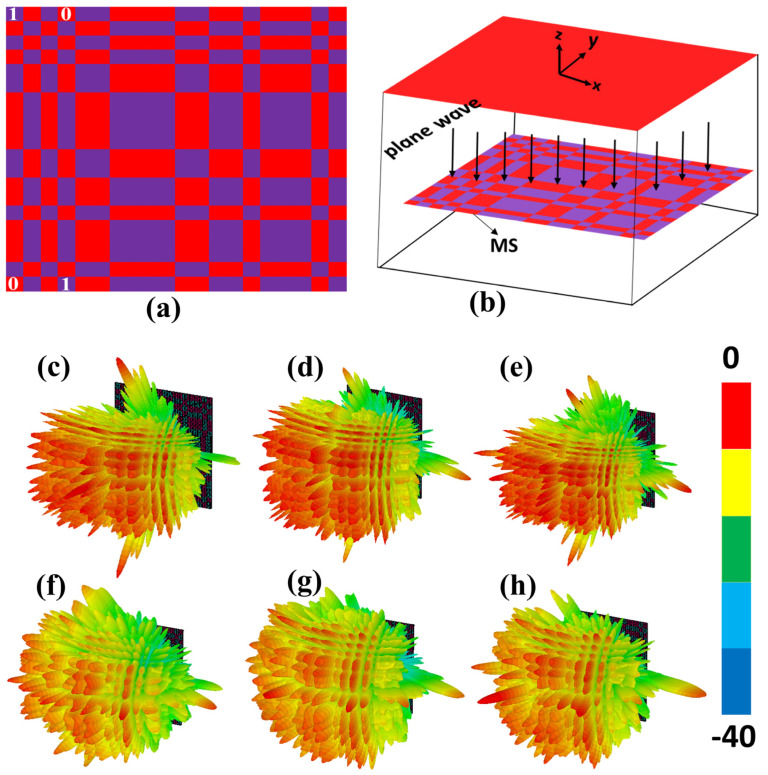
(**a**) Optimized coding sequence of a checkerboard MS. (**b**) Simulation setup. Normalized 3D far-field scattering patterns of a proposed checkerboard MS for (**c**–**e**). TE incidence wave at 34, 36, and 38 GHz. For TM incidence wave at (**f**) 21.5, (**g**) 23, and (**h**) 24.5 GHz.

A checkerboard metasurface is designed to achieve RCS reduction in two different frequency bands, depending on the incidence polarization. To illustrate this phenomenon, we considered a checkerboard MS that consists of *N* × *N* lattices, and each lattice consists of a 3 × 3 subarray of the elements “0” and “1”. The scattering phase of each lattice is assumed to be 0 or π. “10101100001100100010” is the optimized coding sequence of the lattices “0” and “1” that can provide RCS reduction of more than 10 dB whenever the phase difference between “0” and “1” is in the vicinity of π [21]. Coding sequence is similar along vertical and horizontal directions, as shown in Figure 6a. CST Microwave Studio is used to perform full wave simulation of a proposed polarization-dependent checkerboard MS with open add-space boundary conditions in all the directions, as depicted in Figure 6b. Both TE-polarized and TM-polarized plane waves are incident in the −*z*-direction. The distance between the plane wave and a metasurface is set as λ/4. The polarization of a plane wave from TE to TM-polarized is changed by changing the orientation of the E-field. In addition to that, to achieve oblique performance of a MS, a spherical angle of an incident plane wave needs to be changed. For a better understanding of RCS reduction and scattering, far-field 3D patterns of an MS for both polarizations are analyzed. Figure 6c–h illustrates the simulated 3D scattering patterns of a checkerboard MS at 21.5, 23, and 24.5 GHz for TM incident wave and for TE incident wave at 34, 36, and 38 GHz. It can be seen that the incident EM energy is redirected into multiple beams and in all directions, and thus, RCS has been reduced.

## 4. Fabrication and Experimental Results

**Figure 7:**
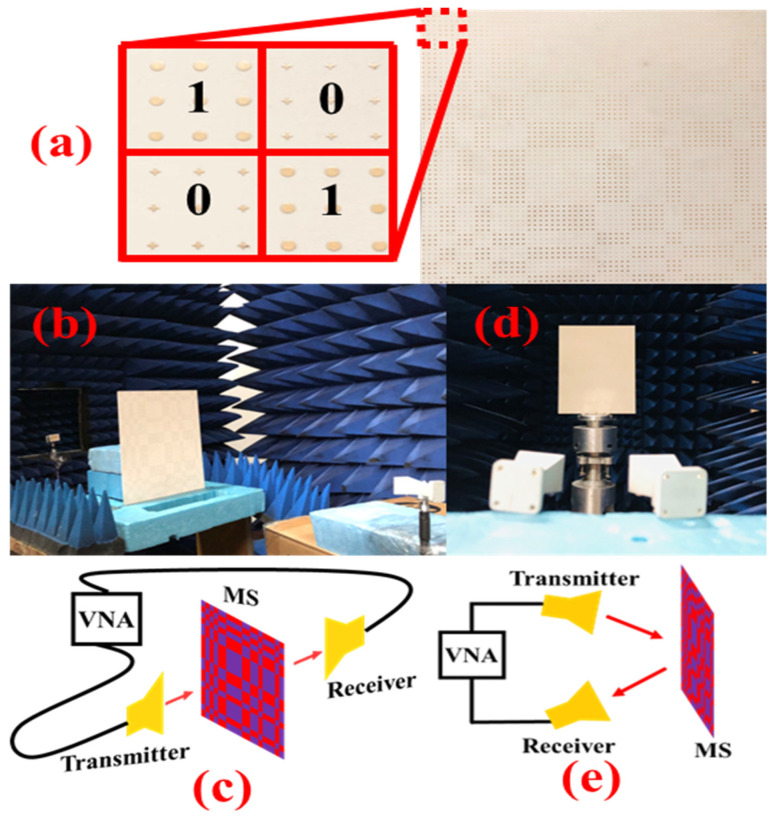
Fabrication and experimental arrangement. (**a**) Fabricated prototype. (**b**,**c**) Experimental and schematic setup of measuring transmission and (**d**,**e**) for reflection.

To further verify the theoretical concept, a prototype of 20 × 20 lattices with a total size 180 × 216 mm^2^ is fabricated, as shown in Figure 7a. All copper metallic plates are etched on a multilayer of a dielectric substrate separately by using the printing circuit board method. Measurements are performed in a shielded chamber to eliminate the noise and reflections from the environment for accurate results of transmission and the backward RCS reduction. Figure 7b,c shows the measurement setup for transmission, while the reflection setup is depicted in Figure 7d,e. As shown in the figures, two broadband horn antennas ranging from 18 to 40 GHz are used to radiate and receive the EM waves that are attached to an Agilent Network Analyzer N5230. Different measurement conditions are obtained by rotation and changing the angles of horn antennas.

**Figure 8:**
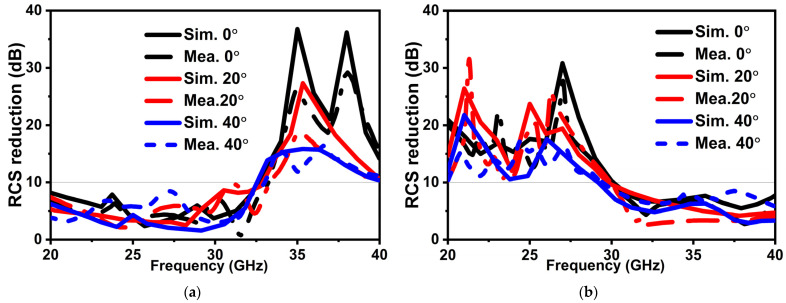
Simulated and measured RCS under (**a**) TE polarization and (**b**) TM polarization with θ = 0°, 20°, and 40°.

**Figure 9:**
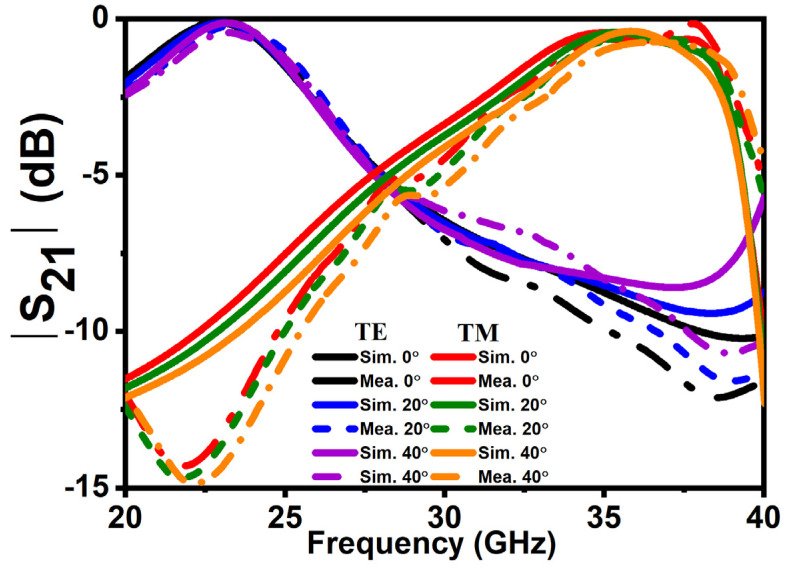
Transmission spectra of a proposed metasurface under normal and oblique TE and TM polarizations.

For backward RCS reduction, two linearly polarized horn antennas are placed on the same side of a coding MS. The RCS reduction performance of a coding MS is calculated by normalizing the reflection coefficients of an MS with an equal-size metallic slab. Figure 8 shows the simulated and measured results for RCS reduction for TE and TM polarizations under normal and oblique incident angles. It is observed that more than 10 dB RCS reduction bandwidth for TE is at *f*2; meanwhile, for TM, it is at *f*1. The overall RCS reduction bandwidth for TE polarization ranges from 33 to 40 GHz, and for TM polarization, it ranges from 20 to 30 GHz, which is coherent according to the phase difference. To realize transmission, first, antennas on the opposite sides of a MS is measured, and then from an equal-size free space. Later, both the measurement results are normalized to achieve transmission properties. Figure 9 shows the transmission performance of the proposed MS. Less than 1 dB insertion loss is observed for TE polarization at 21.1 to 24.5 GHz (*f*1) and similarly for TM polarization at 33.3 to 38.3 GHz (*f*2). The proposed MS has a stable performance up to 40° for both polarizations. In addition to that, a similar response has been achieved on both sides of an interface. A good agreement between simulated and measured results has been observed due to a good experiment environment and a nearly perfect fabricated prototype. However, a slight difference might be caused because of multiple factors, such as (1) tolerance of the substrate at a higher frequency in fabrication and (2) distinction between the dielectric constants used in the simulation and fabrication. Table 1 demonstrates a comparison between the presented work and a previously related published work. Compared with other reported polarization-dependent MS, the advantages of the proposed design are broadband RCS reduction, wide-angle stability, and multiple attributes, which can easily be switched simultaneously between different bands according to actual needs.

## 5. Conclusions

In this paper, a strategy for a passive coding MS to switch functionalities between frequency bands is proposed. Transmission band and reflection band can be interchanged by changing the polarization of an incidence wave. The designed unit cell of a proposed MS consists of a multilayer structure. The results of a proposed MS indicate that above 10 dB, RCS reduction can be achieved at reflection bands of TE and TM polarizations, and less than 1 dB insertion loss in a wide transmission band is achieved at an *f*1 frequency band and an *f*2 frequency band. A checkerboard MS was fabricated for the validation of simulated results. Both simulated and measured results are in good agreement, indicating that the proposed MS has the ability to switch the transmission and reflection wavefronts, depending on the incidence polarization, and achieve wideband of RCS reduction as well. Moreover, the coding MS performance is stable for oblique incidence waves for both horizontal and vertical polarization up to 40°. This research will not only expand the scope of passive MS but also can find its applications in stealth radomes. Besides, this model can easily be implemented to other frequency bands.

## Figures and Tables

**Table 1:** Comparison with state-of-the-art reported work.

Ref	Substrate Layers	Switching Bands	Unit Cell Size (*l* × *w* × *h*) mm	Angular Stability	10 dB RCS Reduction Bandwidth	Mechanism
32	4	1	8 × 8 × 4	-	14 to 15 GHz	Diffusion
33	3	1	11 × 11 × 4.5	-	-	Diffusion
34	2	1	5 × 5 × 2	40°	11.2 to 18.4 GHz	Diffusion
Proposed	3	2	3 × 3 × 2.3	40°	TE = 33 to 40 GHz; TM = 20 to 30 GHz	Diffusion

## Data Availability

The data supporting the findings of this study can be made available to the genuine readers after contacting the corresponding author.
